# Mental health status of people isolated due to Middle East Respiratory Syndrome

**DOI:** 10.4178/epih.e2016048

**Published:** 2016-11-05

**Authors:** Hyunsuk Jeong, Hyeon Woo Yim, Yeong-Jun Song, Moran Ki, Jung-Ah Min, Juhee Cho, Jeong-Ho Chae

**Affiliations:** 1 Department of Preventive Medicine, College of Medicine, The Catholic University of Korea, Seoul, Korea; 2 Department of Cancer Control and Policy, Graduate School of Cancer Science and Policy, National Cancer Center, Goyang, Korea; 3 Department of Psychiatry, Incheon St. Mary’s Hospital, College of Medicine, The Catholic University of Korea, Incheon, Korea; 4 Cancer Education Center, Samsung Comprehensive Cancer Center, Samsung Medical Center, Sungkyunkwan University School of Medicine, Seoul, Korea; 5 Department of Psychiatry, Seoul St. Mary’s Hospital, College of Medicine, The Catholic University of Korea, Seoul, Korea

**Keywords:** Middle East Respiratory Syndrome coronavirus, Isolation, Anxiety, Anger, Korea

## Abstract

**OBJECTIVES:**

Isolation due to the management of infectious diseases is thought to affect mental health, but the effects are still unknown. We examined the prevalence of anxiety symptoms and anger in persons isolated during the Middle East Respiratory Syndrome (MERS) epidemic both at isolation period and at four to six months after release from isolation. We also determined risk factors associated with these symptoms at four to six months.

**METHODS:**

Of 14,992 individuals isolated for 2-week due to having contact with MERS patients in 2015, when MERS was introduced to Korea, 1,692 individuals were included in this study. Anxiety symptoms were evaluated with the Generalized Anxiety Disorder 7-item scale and anger was assessed with the State-Trait Anger Expression Inventory at four to six months after release from isolation for MERS.

**RESULTS:**

Of 1,692 who came in contact with MERS patients, 1,656 were not diagnosed with MERS. Among 1,656, anxiety symptoms showed 7.6% (95% confidence interval [CI], 6.3 to 8.9%) and feelings of anger were present in 16.6% (95% CI, 14.8 to 18.4%) during the isolation period. At four to six months after release from isolation, anxiety symptoms were observed in 3.0% (95%CI, 2.2 to 3.9%). Feelings of anger were present in 6.4% (95% CI, 5.2 to 7.6%). Risk factors for experiencing anxiety symptoms and anger at four to six months after release included symptoms related to MERS during isolation, inadequate supplies (food, clothes, accommodation), social networking activities (email, text, Internet), history of psychiatric illnesses, and financial loss.

**CONCLUSIONS:**

Mental health problems at four to six month after release from isolation might be prevented by providing mental health support to individuals with vulnerable mental health, and providing accurate information as well as appropriate supplies, including food, clothes, and accommodation.

## INTRODUCTION

Since May 20, 2015, when a patient was first diagnosed with Middle East Respiratory Syndrome (MERS) in Korea, the number of patients affected with MERS drastically increased and led to diagnosis of MERS in 186 patients within 45 days, followed by eventual deaths in 38 patients, leaving a record of a 20% mortality rate.

Due to the spread of MERS when preventive vaccine and treatment options were not clearly established, social anxiety and fear caused by uncertainty became core issues. Unverified rumors resulted in sharing of false information.

People concerned about the possibility of being infected with MERS. Some hospitals and schools closed, which increased anxiety in general population. Avoidance of self-isolated individuals with fear of infection of MERS and the tendency to avoid health care workers and their family members became social stigmas and provoked outrage in MERS patients or isolated individuals.

In 2003, the severe acute respiratory syndrome (SARS) epidemic involving 30 countries affected 8,000 individuals, 774 of whom died, causing worldwide concern. At the time, SARS patients experienced social stigma, and reported mental health problems such as anxiety and depression [1]. In an evaluation of mental health status among 1,394 SARS survivors in Hong Kong between 2005 and 2006, 47.8% experienced post-traumatic stress disorder (PTSD) after SARS, and 25.6% of those continued to have PTSD even 30 months after complete treatment for SARS [2].

Mental health evaluation of individuals exposed to natural disasters reveals that survivors typically experience various mental health disorders including PTSD, depression, generalized anxiety disorder, panic disorder, and substance abuse [3-5]. The feeling of anger typically arises in survivors who experience emotional trauma from disasters and this feeling is considered an important factor in regulating the development of PTSD [6-8]. A research study conducted 6-year after the 2003 Daegu subway accident revealed that nearly 40% of victims suffered from PTSD and other difficulties such as avoidance of social relationships, tension, anxiety, and insomnia [9].

There are many research studies evaluating mental health in survivors of natural disasters or infectious diseases. However, no studies have evaluated mental health in individuals isolated due to risk of infection. During the MERS epidemic, more than 80% of the population feared MERS infection [10]. It can be assumed that individuals isolated for 2-week due to being in contact with MERS patients had greater anxiety symptoms and anger such as fear, isolation, and social stigma. With MERS being well-known for a mortality rate of 20%, it is thought that isolated patients had as much fear of being infected as those diagnosed with MERS. However, the effect of 2-week of isolation on the mental health of isolated individuals is not known. We aimed to estimate the prevalence of anxiety symptoms and anger in isolated individuals due to being in contact with MERS both at isolation period and at four to six months after release from isolation. Additionally, we determined the factors associated with these symptoms at four to six months after release.

## MATERIALS AND METHODS

### Subject selection

The data were collected from the epidemiological investigation sector of the center for disease control on patients diagnosed with MERS and isolated individuals who came in contact with MERS patients to perform MERS serum epidemiological investigation. As the research was conducted as an additional epidemiological investigation, individuals with high risk of positive serum results were selected from those who came in contact with MERS patients to participate in this study. Four regions with high prevalence of MERS patients including Seoul, Gyeonggi, Chungcheong, and Gangwon were selected. In each region, study subjects were selected in the order of higher likelihood of MERS diagnosis. The ranking order was as follows: 0 rank for MERS diagnosed patients, 1st rank for partners, the same hospital patients, caregivers or visitors of MERS diagnosed patient with extreme likelihood of spreading disease, 2nd rank for partners, the same hospital patients, caregivers or visitors of MERS diagnosed patient with likelihood of spreading disease, 3rd rank for partners, the same hospital patients, caregivers or visitors of MERS diagnosed patients, and 4th rank for random individuals who came in contact with MERS diagnosed patients. The data provided included individuals with unverified contact information that prevented them from being ranked.

A diagnosed patient was defined as a patient with MERS coronavirus that was verified in a laboratory diagnostic test. A patient with positive contact was defined as an individual who, without wearing appropriate self-protective equipment such as gown, gloves, N95 mask, goggles or face mask, stayed within 2 m of a MERS patient, stayed in the same room or the ward as a MERS patient, or came in direct contact with respiratory secretions of a MERS patient [11]. Since the incubation period of MERS is between two to 14 days, patients who came in contact should be monitored for occurrence of symptoms for at least 14 days. Individuals who were verified to have direct contact during the period of 14 days were isolated for 2-week in the house, workplace, and hospital.

**Figure 1.**
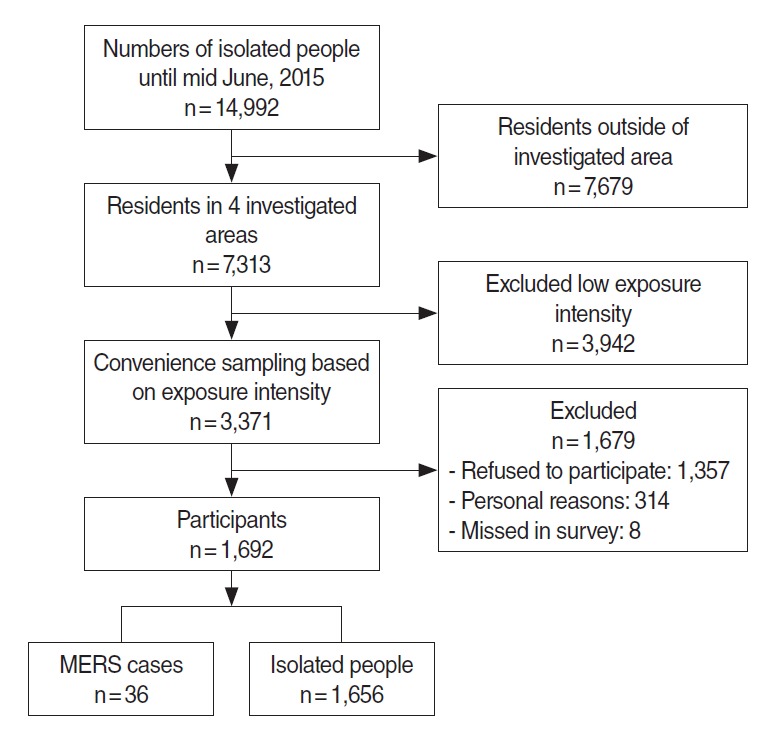
Flow diagram of selection of the participants. MERS, Middle East Respiratory Syndrome.

A list of 14,992 individuals reported to have been isolated from the end of May to mid-June of year 2015 was provided by the Department of Epidemiologic Investigation of the Centers for Disease Control and Prevention. Of those individuals, calls requesting interest in research participation were made to 3,371 individuals out of 7,313 residents living in target regions (Seoul, Gyeonggi, Chungcheong, and Gangwon). Subject selection was prioritized to partners, same hospital patient, caregivers, and visitors of MERS patients residing in the target regions. A total of 1,692 individuals (50.0%) agreed to participate in this study, and 1,679 individuals refused. Of those who refused to participate, 65 individuals (4.8%) showed strong refusal to participate in the study with profanity and ranting, 315 individuals (23.3%) ranted in refusal to participate, and 568 individuals (41.9%) responded with simple refusal. Four hundred nine individuals (30.0%) had difficulties preventing them from participating in the study and reasons for refusal included the following: death in the family, nursing, inpatient hospitalization and surgery, work, opposition from the family, argument for inadequate qualification as a study subject, and reluctance to be known as a study subject. Three hundred fourteen individuals could not participate in the study for personal reasons, and eight individuals were not studied. Of 1,692 individuals included in the study, 36 individuals were diagnosed with MERS during isolation, and 1,656 individuals were not definitively diagnosed with infection (Figure 1). This study received approval from the bioethics committee of the Centers for Disease Control and Prevention (2015-07-EXP-01-R-A) and the institutional review board of the National Cancer Center (NCC2016-0058).

### Procedures

Mental health evaluation was performed by a trained researcher asking survey questions and recording the subject’s answers. The survey took place at the community health centers, along with a serology test. Surveys were conducted from September to November 2015, and a time point four to six months after removal from isolation for MERS.

### Measurements

A retrospective survey was done the followings: MERS related symptoms such as fever, cough, and diarrhea during the isolation period, availability of food, clothing, and water, possibility of bathing, availability of living necessities, the use of phone calls, texts or email, Internet, and the state of isolation such as at the hospital, alone at home/hotel, or with family at home. General information such as sex and occupation, history of psychiatric illnesses, history of medical problems, medical expenses for MERS, and details on financial loss were investigated. Symptoms of anxiety and anger at the time of isolation were measured retrospectively, and they were also measured at four to six months after removal of isolation.

#### Anger

Anger was evaluated by Korean version of the State-Trait Anger Expression Inventory (STAXI) derived from the original version STAXI by Spielberger [12,13]. This scale is consisted of 10-item with 4-point Likert scale that ranged from 1 (“not at all”) to 4 (“almost always yes”), with higher scores referring to higher likelihood. Total scores ranged from 10 to 40 points. In this study, the cut-off for anger was decided on a total score of 14 points. Cronbach’s alpha was 0.94 in this study.

#### Anxiety

Anxiety was assessed by 7-item Generalized Anxiety Disorder Scale (GAD-7). GAD-7 is a self-administered test to assess generalized anxiety disorder that is composed of 7-item highly relevant questions selected from a total of 13 items (nine questions from the Diagnostic and Statistical Manual of Mental Disorder, 4th ed. and four questions from the Anxiety Symptom Scale) [14]. For each of 7 items, subjects were asked about how frequently they felt each one during the isolation period for MERS and again four to six months after removal from isolation. The 4-point Likert scoring system was used as follows: not at all (0 point), several days (1 point), more than half the days (2-point), nearly every day (3-point). Higher scores implied greater anxiety symptoms. The total score ranged from o to 21, with a score range of 5-9 points classified as mild anxiety symptoms, 10-14 points as moderate anxiety symptoms, and greater than 15 points as severe anxiety symptoms. In the present study, a total score greater than or equal to 10 points was set as a cut-off score for moderate anxiety symptoms and individuals with that score were categorized into the anxiety group. In the study, Cronbach’s alpha was 0.95.

### Statistical analysis

From the isolated individuals, those diagnosed with MERS were classified as MERS patients, and those not diagnosed with MERS were named isolated patients.

General information and the state of isolation as well as living environment during isolation were presented for each group (MERS patients and isolated patients) in frequencies and percentages. Symptoms of anxiety and anger during isolation and four to six months after removal from isolation were presented in frequencies and percentages for each group. The two groups’ differences were analyzed with chi-square tests. Protective factors and risk factors for experiencing symptoms of anxiety and anger at four to six months after removal from isolation were presented as relative risk (RR) with 95% confidence interval (CI) adjusted for sex and age using the PROC GENMOD of SAS (SAS Institute Inc., Cary, NC, USA). Chi-square test was used to assess whether there were any significant differences in the frequency of anxiety and anger symptoms among 1,023 subjects (60%) with validated data depending on the likelihood of contact, because investigation in this study prioritized individuals with a high likelihood of contact with MERS patients. Since subjects might have refused participation out of anger, we analyzed subjects’ reason for refusing participation to determine if the prevalence of the participants’ anxiety symptoms and anger was underestimated. All statistical analyses were performed with SAS version 9.3 (SAS Institute Inc., Cary, NC, USA), and *p*-values less than or equal to 0.05 were considered statistically significant.

## RESULTS

When it comes to isolated people were as follows: 944 (57.0%) females, 795 (48.0%) jobless people, 33 (2.0%) with history of psychiatric illness, 375 (22.6%) with history of physical illness, 195 (11.8%) people reported medical expenses due to MERS, and 173 (10.5%) people reported financial losses and decrease in sales. The most common infection route was patients from the same hospital (33.3%), then visitors (26.7%), hospital workers (16.1%), and patient’s family members (9.9%) (Table 1).

In isolated people, 8.5% experienced fever, 7.0% cough, and 4.1% diarrhea. Compared to isolated people, MERS patients experienced more symptoms related to MERS such as fever, cough, and diarrhea (p<0.001). Eighty seven point three percent of isolated people reported having enough food and water. Ninety six point six percent of isolated people could bathe during the isolation period, and 97.0% of isolated people felt equipped with daily necessities. Compared to MERS patients, isolated people received more food and shelter support (p<0.001). There was no significant difference between MERS patients and isolated people in social activity such as phone calls, texts, email, or Internet use. For isolation location, 91.7% of MERS patients stayed in the hospital and 8.3% stayed with family at home, whereas 68.4% of isolated people stayed with family at home and 6.3% stayed in the hospital. This revealed a statistical difference in the location of isolation between MERS patients and isolated people (p<0.001) (Table 2).

During isolation, 47.2% (95% CI, 30.9 to 63.5%) of MERS patients had symptoms of anxiety and 52.8% (95% CI, 36.5 to 69.1%) had feelings of anger. Four to six months after removal from isolation, 19.4% (95% CI, 6.5 to 32.3%) of MERS patients had symptoms of anxiety and 30.6% (95% CI, 15.6 to 45.7%) had feelings of anger. In isolated people during isolation, 7.6% (95% CI, 6.3 to 8.9%) had symptoms of anxiety and 16.6% (95% CI, 14.8 to 18.4%) had feelings of anger. Four to six months after removal from isolation, 3.0% (95% CI, 2.2 to 3.9%) had symptoms of anxiety and 6.4% (95% CI, 5.2 to 7.6%) had feelings of anger (Table 3).

After adjusting for sex and age, risk factors for experiencing anxiety symptoms and anger at four to six months after release included symptoms related to included symptoms related to MERS during isolation, inadequate supplies (food, clothes, accommodation), social networking activities (email, text, Internet), history of psychiatric illnesses, and financial loss (Table 4).

**Figure 2.**
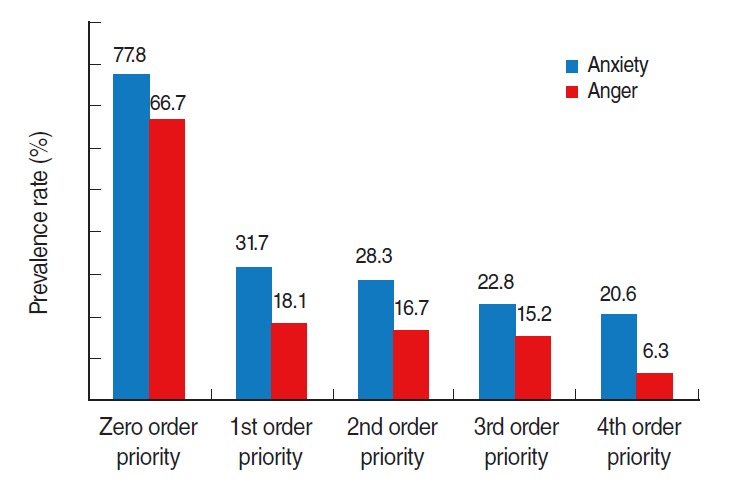
The prevalence rate of anxiety symptoms and anger according to the exposure intensity of Middle East Respiratory Syndrome.

In 1,032 people with verified ranks, 27 people were in the 0th rank (2.6%), 514 in the 1st rank (49.8%), 60 in the 2nd rank (5.8%), 368 in the 3rd rank (35.7%), and 63 in the 4th rank (6.1%). On analysis of prevalence differences of anxiety symptoms and anger based on the likelihood of exposure (prioritized in ranking system), the prevalence rate of anger increased with priority in rank evidence by the following distribution: 18.1% in the 1st rank, 16.7% in the 2nd, 15.2% in the 3rd, and 6.3% in the 4th (p for trend<0.001). The prevalence rate of anxiety symptoms in each rank were as follows: 31.7% in the 1st rank, 28.3% in the 2nd, 22.8% in the 3rd, and 20.6% in the 4th (p for trend<0.001), which revealed that the prevalence rate of anxiety symptoms increased with priority in rank (Figure 2).

## DISCUSSION

The incubation period of MERS is an average of five days (2-14 days), so symptoms typically occur a minimum of two days or a maximum of 14 days after exposure to the MERS virus. Thus, people who have been in close contact should be monitored closely for at least 14 days for occurrence of symptoms. As of June 10, 2015, amid the MERS epidemic, people were isolated in 17 cities nationwide. There were nearly 500 people in isolation in areas where there was a major outbreak, and entire villages in some areas were isolated. At that time, the number of people isolated at home reached 3,000 nationwide, and there was a trend increasing by 200 to 300 people per day. With the rapid spread of MERS into Korean society, it is likely that people who were to be isolated had fears of infection and anxiety over MERS which had over a 20% mortality rate, concern over social isolation, and anxiety over the possibility of spreading infection to family members if isolated at home. Since community health care center staff members were monitoring the state of isolation to prevent MERS spreading further, it is likely that those isolated had high levels of anxiety over the fear of their isolation becoming a stigma among their neighbors. During the MERS epidemic, 80.2% of the general public reported fear of being infected, and 46% reported emotional distress. Risk factors associated with increased rates of fear were the following: concern about public transportation use, difficulty going outside, perception that the state is not protecting the people, helplessness in situations that cannot be controlled, and fear of infection [10].

During isolation, symptoms of anxiety were reported in 47.2% of MERS patients and 7.6% of quarantined people. At the time point four to six months after removal from isolation, symptoms of anxiety persisted in 19.4% of MERS patients and 3.0% of isolated people. Anxiety symptoms were determined based on 10 or more of the GAD-7 score were determined to have anxiety symptoms. A moderate range of anxiety symptoms is defined as having difficulty in daily life due to anxiety for an average of 10.7 to 16.8 days out of a 3-month period, as well as having 2.2 to 2.4 hospital visits due to anxiety symptoms [14]. When the GAD-7 cut-off point is set as 10 points or higher, the prevalence of anxiety symptoms in the general population is estimated to be around 3.3%. This is much lower than the prevalence rate of anxiety disorder (6.8%) using the Composite International Diagnostic Interview of the nationwide mental health epidemiologic survey in 2011 [15]. This suggests that there is a possibility of underestimating the actual prevalence of anxiety symptoms when the GAD-7 cut-off point is set as 10 points or higher. When anxiety symptoms in the Ansan and Gyeonggi areas were measured by the GAD-7 four to six months after the sinking of the passenger ship on April 16, 2014, 6.4% of residents in the Ansan area and 3.3% in the Gyeonggi area reported moderate to severe levels of anxiety symptoms [16]. When comparing these results, isolated people appear to have recovered to normal levels of anxiety four to six months after removal from isolation. However, among 19% of those in the 1st rank group who had anxiety symptoms during isolation continued to have anxiety symptoms even four to six months after removal from isolation. Thus, it is difficult to say that isolation-induced anxiety has normalized.

The rate of anger was 16.6% during isolation, and 6.4% around four to six months after isolation. The state of anger is measured by assessing the temporary feeling of anger experienced under stress by a person who does not have anger disorder. It is a temporary state that can change with time, as the problem causing stress is resolved.

Anger status was defined as a STAXI total score of 14 or higher, which is equivalent to the average score of a prison inmate with moderate to severe symptoms of depression (28.1%), suicidal thoughts (33.6%), and alcohol abuse (39.1%) [17].

Of people isolated for MERS with symptoms of anger, 28.1% had persistent anger even four to six months after removal of isolation. Of those with 1st rank group, 50% had sustained anger four to six months after removal of isolation. Although the prevalence of anger could have been overestimated in this study because the participants prioritized as individuals ranked first according to the ranking system of MERS exposure. However, the prevalence of anger in isolated people might have been underestimated considering that nearly 30% of individuals who did not participate in the study expressed anger through cursing or profanity.

At the time of isolation, the Korean Red Cross and local public health centers provided rice, water, ramen, and daily necessities that can be used by families of four for a week.

However, in some cases, not all people were provided with relief items at the time of isolation, so it seems likely that anxiety symptoms and anger were largely felt when the necessary supplies for daily life were not provided at appropriate times. Approximately 50% of those isolated for MERS received relief supplies; however, 98% of the study participant reported that they received relief supplies. This discrepancy is likely due to inclusion of subjects with higher priority in the survey.

In patients with history of psychiatric illnesses, there was a high risk of anxiety and anger at four to six months after removal from isolation. These patients seem to have weak control of anxiety and anger symptoms related to the emotional center, because psychiatric illnesses involve neurotransmitter abnormalities in the cerebrum [18]. This suggests that special interventions are necessary for people with a history of psychiatric illness in traumatizing situations.

Progression of anxiety symptoms, anger, and aggression experienced in the early stages of natural disaster can be prevented by early mental health care [19]. However, without early intervention, these symptoms evolve into long-term PTSD. Thus, symptoms of anxiety and anger should be recognized early on and appropriate intervention needs to be implemented for improvement in symptoms in the short-term.

During the isolation period, relief supplies must be provided on a timely basis. Precise information about the symptoms of the disease should be provided publicly, and psychological support is needed in those with persistent symptoms even after removal of isolation. Any financial losses should be identified and properly supported. Psychological support is necessary in those with history of psychiatric illnesses as they have a greater likelihood of experiencing psychiatric symptoms. Medical management programs should be provided in patients with persistent symptoms.

Since anxiety symptoms, anger, and aggression due to natural disasters can be prevented from becoming chronic problems with appropriate mental health management, progression to PTSD should be prevented by providing prompt responses, meeting individualized needs, and making efforts to provide appropriate intervention after identifying symptoms of anxiety and anger.

People who were isolated for two weeks due to contact with MERS patients suffered from high rates of anxiety symptoms and anger during isolation, and showed mental health effects even at four to six months after removal from isolation.

## Figures and Tables

**Table 1.** General characteristics of 1,692 MERS-cases and isolated people

	MERS cases (n = 36)	Isolated people (n = 1,656)	p-value
Sex			0.40
Male	18 (50.0)	712 (43.0)	
Female	18 (50.0)	944 (57.0)	
Age (mean±SD)	52.3±15.0	43.9±19.2	0.009
Job			0.47
Self-employed	6 (16.7)	174 (10.5)	
Employed	13 (36.1)	687 (41.5)	
Unemployed	17 (47.2)	795 (48.0)	
History of mental disease			< 0.001
Yes	8 (22.2)	33 (2.0)	
No	28 (77.8)	1,623 (98.0)	
History of physical disease			0.02
Yes	14 (38.9)	375 (22.6)	
No	22 (61.1)	1,281 (77.4)	
Medical cost expenditure			< 0.001
Yes	20 (55.6)	195 (11.8)	
No	16 (44.4)	1,461 (88.2)	
Financial loss			0.23
Yes	6 (16.7)	173 (10.5)	
No	30 (83.3)	1,483 (89.5)	
Infection routes			< 0.001
Same ward patients	20 (55.7)	551 (33.3)	
Patients’ family	0 (0.0)	164 (9.9)	
Care takers	6 (16.7)	30 (1.8)	
Visitor	4 (11.1)	442 (26.7)	
Hospital workers	4 (11.1)	267 (16.1)	
Others	2 (5.7)	202 (12.2)	

Values are presented as number (%).

MERS, Middle East Respiratory Syndrome; SD, standard deviation.

**Table 2.** Living status and environmental situation during isolation due to Middle East Respiratory Syndrome (MERS) exposure

Variables	MERS cases (n = 36)	Isolated people (n = 1,656)	p-value
MERS related symptoms			< 0.001
Fever	28 (77.8)	141 (8.5)	
Cough	20 (55.6)	116 (7.0)	
Diarrhea	16 (44.4)	67 (4.1)	
Food, clothes, and house supplies			< 0.001
Sufficient food and water	23 (63.9)	1,446 (87.3)	
Able to bathe	27 (75.0)	1,600 (96.6)	
Having self-care products	29 (80.6)	1,606 (97.0)	
Social networking activities			
Making phone calls	31 (86.1)	1,354 (81.8)	0.50
Texting or e-mailing	1 (2.8)	111 (6.7)	0.51
Using the Internet	2 (5.6)	231 (13.9)	0.22
Isolation environment			< 0.001
Hospital	33 (91.7)	104 (6.3)	
Alone (in home or hotel)	0 (0.0)	419 (25.3)	
With family	3 (8.3)	1,133 (68.4)	

Values are presented as number (%).

**Table 3.** Prevalence of anxiety symptoms and anger during isolation and 4-6 months after release

Variables	MERS cases (n = 36)	Isolated people (n = 1,656)
In isolation		
Anxiety symptoms	47.2 (30.9, 63.5)	7.6 (6.3, 8.9)
Anger	52.8 (36.5, 69.1)	16.6 (14.8, 18.4)
4-6 months after release		
Anxiety symptoms	19.4 (6.5, 32.3)	3.0 (2.2, 3.9)
Anger	30.6 (15.6, 45.7)	6.4 (5.2, 7.6)

Values are presented as relative risk (95% confidence interval). MERS, Middle East Respiratory Syndrome.

**Table 4.** Relative risks^1^ of anxiety symptoms and anger at 4-6 months after release from isolation by the factors during the isolation period

Variables (reference)	MERS cases (n = 36)		Isolated people (n = 1,656)	
	Anxiety (n = 7)	Anger (n = 11)	Anxiety (n = 49)	Anger (n = 106)
MERS related symptoms				
Fever (no)	1.7(0.3, 9.1)	1.5 (0.3, 9.2)	1.8 (1.1, 3.0)	2.4 (1.4, 4.1)
Cough (no)	1.8 (0.4, 7.7)	1.6 (0.4, 6.9)	3.1 (2.0, 5.0)	3.8 (2.3, 6.3)
Diarrhea (no)	3.7 (0.8, 18.0)	4.1 (0.8, 20.6)	5.3 (3.1, 9.0)	6.7 (3.8, 11.8)
Food, clothes, and house supplies				
Sufficient food and water (yes)	5.9 (0.9, 34.5)	3.8 (0.8, 18.6)	3.2 (2.2, 4.7)	3.2 (2.0, 5.0)
Able to bathe (yes)	3.3 (0.6, 17.8)	1.1 (0.2, 5.7)	2.1 (1.1, 4.2)	2.9 (1.3, 6.4)
Having self-care products (yes)	1.0 (0.2, 5.9)	2.1 (0.4, 12.4)	2.7 (1.3, 5.3)	3.1 (1.4, 6.7)
Social networking activities				
Making phone calls (no)	1.3 (0.1, 13.6)	1.7 (0.1, 21.4)	1.1 (0.7, 1.7)	1.1 (0.7, 1.8)
Texting or e-mailing (no)	NA	NA	2.2 (1.3, 3.7)	2.2 (1.2, 4.0)
Using Internet (no)	NA	NA	1.8 (1.1, 2.7)	1.9 (1.1, 3.1)
History of mental disease (no)	10.7 (1.1, 109.6)	2.9 (0.5, 17.3)	5.3 (2.5, 11.0)	4.0 (1.7, 9.5)
History of physical disease (no)	2.4 (0.6, 10.2)	3.4 (0.7, 15.6)	1.8 (1.3, 2.6)	1.5 (0.9, 2.4)
Medial cost expenditure (no)	5.5 (1.0, 30.7)	1.6 (0.3, 7.7)	3.7 (2.5, 5.5)	5.5 (3.5, 8.5)
Financial loss (no)	3.3 (0.7, 15.7)	1.2 (0.3, 5.6)	1.9 (1.4, 2.6)	1.6 (1.1, 2.3)

Values are presented as relative risk (95% confidence interval). MERS, Middle East Respiratory Syndrome; NA, not available.

1 Adjusted for age and sex.

## References

[1] Chua SE, Cheung V, Cheung C, McAlonan GM, Wong JW, Cheung EP (2004). Psychological effects of the SARS outbreak in Hong Kong on high-risk health care workers. Can J Psychiatry.

[2] Mak IW, Chu CM, Pan PC, Yiu MG, Ho SC, Chan VL (2010). Risk factors for chronic post-traumatic stress disorder (PTSD) in SARS survivors. Gen Hosp Psychiatry.

[3] Acierno R, Ruggiero KJ, Galea S, Resnick HS, Koenen K, Roitzsch J (2007). Psychological sequelae resulting from the 2004 Florida hurricanes: implications for postdisaster intervention. Am J Public Health.

[4] Mason V, Andrews H, Upton D (2010). The psychological impact of exposure to floods. Psychol Health Med.

[5] Norris FH (2005). Range, magnitude, and duration of the effects of disasters on mental health: review update 2005. Res Educ Disaster Ment Health.

[6] Galea S, Nandi A, Vlahov D (2005). The epidemiology of post-traumatic stress disorder after disasters. Epidemiol Rev.

[7] Evans S, Giosan C, Patt I, Spielman L, Difede J (2006). Anger and its association to distress and social/occupational functioning in symptomatic disaster relief workers responding to the September 11, 2001, World Trade Center disaster. J Trauma Stress.

[8] Jakupcak M, Conybeare D, Phelps L, Hunt S, Holmes HA, Felker B (2007). Anger, hostility, and aggression among Iraq and Afghanistan War veterans reporting PTSD and subthreshold PTSD. J Trauma Stress.

[9] Lee SY (2011). A study on the life experiences of the victims’ families and the realities of related agencies of managing the disaster in the Daegu subway. Korean J Soc Welf Educ.

[10] Lee DH, Kim JY, Kang HS (2016). The emotional distress and fear of contagion related to Middle East Respiratory Syndrome (MERS) on general public in Korea. Korean J Psychol Gen.

[11] Korea Centers for Disease Control and Prevention MERS. http://www.mers.go.kr/mers/html/jsp/main.jsp.

[12] Spielberger CD, Gorsuch RL, Lushene RE (1983). STAI manual for the state-trait anxiety inventory: self-evaluation questionnaire.

[13] Chon KK, Hahn DW, Lee CH, Spielberger CD (1997). Korean adaptation of the State-Trait Anger Expression Inventory: anger and blood pressure. Korean J Health Psychol.

[14] Spitzer RL, Kroenke K, Williams JB, Löwe B (2006). A brief measure for assessing generalized anxiety disorder: the GAD-7. Arch Intern Med.

[15] Ministry of Health and Welfare (2011). Survey on mental dynamics, 2011.

[16] Yang HJ, Cheong HK, Choi BY, Shin MH, Yim HW, Kim DH (2015). Community mental health status six months after the Sewol ferry disaster in Ansan, Korea. Epidemiol Health.

[17] Park JI, Kim YJ, Lee SJ (2013). Mental health status of prisoners in correctional institutions. J Korean Neuropsychiatr Assoc.

[18] Yehuda R (2002). Current status of cortisol findings in post-traumatic stress disorder. Psychiatr Clin North Am.

[19] Adams RE, Boscarino JA, Galea S (2006). Social and psychological resources and health outcomes after the World Trade Center disaster. Soc Sci Med.

