# Supplementary material for: Mental health status of people isolated due to Middle East Respiratory Syndrome
Source: Epidemiol Health. 2016 Nov 5;38:e2016048. doi: 10.4178/epih.e2016048 (PMC5177805; doi:10.4178/epih.e2016048)
Supplement: Supplementary file 1 [file epih-38-e2016048-supplementary.pdf]

메르스 격리자에서의 정신건강

Mental health status of the isolated people due to MERS

정현숙[1], 임현우[1*], 송영준[2], 기모란[2], 민정아[3], 조주희[4], 채정호[5]

Hyunsuk Jeong[1], Hyeon Woo Yim, [1*], Yeong-Jun Song[2], Moran Ki[2], Jung-Ah Min[3], Juhee Cho[4], Jeong-Ho Chae[5]

1. Department of Preventive Medicine, College of Medicine, The Catholic University of Korea, Seoul, Korea
2. Department of Cancer Control and Policy, Graduate School of Cancer Science and Policy, National Cancer Center, Goyang, Korea
3. Department of psychiatry, Incheon St. Mary's Hospital, The Catholic University of Korea, College of Medicine, Incheon, Korea
4. Cancer Education Center, Samsung Comprehensive Cancer Center, Samsung Medical Center, Sungkyunkwan University School of Medicine, Seoul, Korea
5. Department of psychiatry, Seoul St. Mary's Hospital, The Catholic University of Korea, College of Medicine, Seoul, Korea

Correspondence:

Name: Hyeon Woo Yim, MD, PhD

Address: Department of Preventive medicine, College of Medicine, The Catholic University of Korea. Banpodero #222, Seochogu, Seoul Korea

Tel: +82-2-2258-7860

Fax: +82-2-532-3820

E-mail: y1693@catholic.ac.kr

Acknowledgements:

본 연구는 질병관리본부 연구용역과제의 연구비를 지원받아 수행되었습니다. (연구사업번호: 2015P7400300)

Conflict of interest

No Disclosure to Report

ORCID ID:

Hyunsuk Jeong: 0000-0001-5274-3816

Hyeon Woo Yim: 0000-0002-3646-8161

Abstract

Background and purpose:

감염병 관리로 인한 격리는 정신건강에 영향을 줄 것으로 여겨지나 아직까지 그 영향에 대해서 알려진바 없다. 메르스 유행으로 격리되었던 사람에서 격리 당시의 불안증상과 분노감 유병율을 알아보고 격리해제 후 4-6개월 시점에 불안증상과 분노감 유병율과 그 위험요인을 파악하고자 하였다.

Methods:

2015 년 한국에 메르스 유입으로 메르스 환자와의 밀착접촉으로 2 주간 격리조치 된 14,999 명 중 1,692 명을 대상으로 메르스 격리해제 후 4-6 개월 지난 시점에서 GAD-7 을 이용하여 불안증상을 평가하였고 STAI 를 이용하여 분노감을 평가하였다. 격리 당시의 불안증상과 분노감, 그리고 위험요인은 후향적으로 평가하였다.

Results:

밀착접촉자 1,692명 중 1,656명은 메르스로 진단되지 않았다. 격리기간동안 격리자의 7.6% (95%CI: 6.3-8.9)가 불안증상이 있었고, 16.6% (95%CI: 14.8-18.4)가 분노감이 있었다. 격리해제 후 4-6개월 지난 시점에서 격리자의 3.0% (95%CI: 2.2-3.9)가 불안증상이 있었고, 6.4% (95%CI: 5.2-7.6)가 분노감이 있었다. 격리해제 후 4-6개월 지난 시점에서 불안증상 및 분노감을 경험하는 위험요인으로는 격리 당시 메르스 증상이 있었거나 구호품 보급이 충분하지 않았거나 이메일, 문자, 인터넷 등을 사용하였거나 정신과 질환의 과거력이 있었던 경우, 재정 손실이 있었던 사람이었다.

Conclusion:

격리해제 후 4-6개월 시점에 정신건강문제는 정신건강에 취약한 사람에 대한 심리지원, 격리 기

간 동안 정확한 정보제공과 시의 적절한 구호품을 제공함으로써 예방 할 수 있을 것이다.

Key words: MERS, Isolation, Anxiety, Anger

서론

2015 년 5 월 20 일 한국에서 중동호흡기증후군 (Middle East Respiratory Syndrome, MERS; 메르스) 첫 확진자가 발생한 이후 환자가 급속도로 증가하여 45 일만에 186 명의 확진 환자가 발생하였고 이 중 38 명이 사망하여 치사율이 20%라는 기록을 남겼다.

예방 백신과 치료제가 확립되지 않은 상황에서 메르스 감염이 확산되었기 때문에 불확실성에 기인하는 사회적인 불안증상과 두려움이 핵심이슈가 되었고, 검증되지 않은 유언비어와 루머로 인한 잘못된 정보들이 공유되기도 하였다.

사람들은 메르스 감염 가능성에 대해 걱정을 하게 되었고, 일부 병의원 폐쇄조치가 이루어지기도 하였고 학교의 휴교까지 이어지면서 사회 일반에 불안감이 증가했다. 전염성 질환 특성 때문에 자가격리 해제된 대상자를 기피하거나 메르스 발생 병의원에 종사하는 의료인 및 가족들을 멀리하는 경향은 사회낙인으로 번지게 되어 메르스 확진자나 격리자에게 분노감을 유발하도록 하였다. 2003 년 30 개국에 걸쳐 대 유행하였던 SARS(severe acute respiratory syndrome) 로 인해 8,000 명의 감염자가 발생하였고, 이 중 774 명이 사망하여 전 세계에 불안감을 주었었다. 당시 SARS 감염자는 사회적 낙인을 경험하였고 불안증상, 우울증 등 정신건강 문제를 호소하였다 [1]. 2005-2006 년 홍콩에서 SARS 생존자 1,394 명을 대상으로 정신건강을 평가한 결과, 47.8%가 SARS 이후에 외상 후 스트레스장애 (PTSD)를 경험하였고, 이 중 25.6%는 30 개월이 지난 시점에서도 정신건강에 문제를 호소하고 있어서 정신적인 후유증이 장기화 되는 것으로 나타났다 [2].

자연재해에 노출되었던 사람들의 정신건강평가에서 재난 생존자들은 외상 후 스트레스장애를 비롯하여 우울증, 범불안장애, 공황장애, 물질남용 등 다양한 정신질환을 겪는 것으로 나타났다 [3-5]. 재난으로 인해 정신적 충격을 경험한 생존자들에서 분노 감정은 일반적으로 생겨나는 것으로

외상후 스트레스장애의 발달과 조절에 있어서 중요한 요인으로 주목받고 있다 [6-8]. 2003 년 국내에서 발생한 대구지하철 사고 6 년 후 조사한 연구에 따르면 40%에 달하는 피해자들이 외상후 스트레스장애를 겪고 있었고 사회적 관계를 회피하거나 긴장감, 불안감, 수면문제 등의 어려움을 겪고 있는 것으로 나타났다 [9].

자연재해, 인재, 혹은 감염성질환으로 인한 생존자들에 대한 정신건강평가 연구는 많이 이루어지고 있으나 감염위기로 격리되었던 사람들에 대한 정신건강 평가는 아직까지 보고된 바 없다. 메르스 당시 일반 인구의 80% 이상이 메르스 감염에 대한 두려움이 있었던 것으로 나타났는데 [10] 메르스 확진자와의 밀착접촉으로 인해 2 주간의 격리를 경험했던 메르스 격리자의 경우 메르스 감염에 대한 두려움과 고립감, 사회적 낙인 등으로 불안증상과 분노감이 컸을 것으로 여겨진다. 특히 치명율이 20%에 달하는 것으로 알려져 있었기 때문에 격리자들은 전염병에 걸릴 지 모른다는 불안증상이 실제 감염자만큼이나 있었을 것으로 생각된다. 하지만 아직까지 2 주간의 격리가 격리자의 정신건강에 미치는 영향에 대해서는 아직까지 알려진바 없다. 본 연구는 메르스환자와의 밀착접촉으로 격리되었던 사람에서 격리 당시의 불안증상과 분노감이 어떠했는지를 알아보고 격리해제 후 4-6 개월 시점에 불안증상과 분노감 유병율을 파악 하고자 하였다. 또한 격리 해제후 4-6 개월 시점에 불안증상과 분노감 유병과 연관된 격리기간동안의 요인을 파악하고자 하였다.

방법

대상자선정

본 조사는 메르스 혈청학적 역학 조사를 수행하기 위해 질병관리본부 역학조사과에서 구축한 메르스 확진자와 메르스 환자 접촉으로 인한 격리자 데이터를 제공받아서 수행하였다. 혈청학적 역학조사의 부가적인 연구로 수행되었기 때문에 대상자 선정에 있어서 메르스 환자 접촉으로 인한 격리자 중 혈청학적으로 양성 가능성이 높은 고위험 집단을 선정하였다. 메르스 환자가 많이 발생한 서울, 경기, 충청, 강원 등 4 개 지역을 선정하였다. 각 지역내에서 조사 대상자 선정은 격리자 중에서 확진가능성이 높은 순서로 조사대상자를 선정하였다. 확진가능성에 따른 순위는 0 순위는 확진환자, 1 순위는 슈퍼전파 확진환자의 동거인, 같은 병원환자, 간병/방문객으로 구성되어 있었고, 2 순위는 감염전파 확진환자의 동거인, 같은 병원환자, 간병/방문객이었고, 3 순위는 그 외 확진환자의 동거인, 같은 병원환자, 간병/방문객이었으며 4 순위는 확진환자의 일반인 접촉자로 정의하였다. 제공 받은 자료에서 접촉 관련 정보가 미확인되어 순위를 부여할 수 없는 사람도 있었다.

확진환자의 정의는 실험실 진단검사를 통해 메르스 코로나바이러스 감염이 확인된 사람이었다.

접촉자의 정의는 적절한 개인보호장비인 가운, 장갑, N95 마스크, 고글 또는 안면보호구 등을 착용하지 않고 메르스 환자와 2 미터 이내에 머문 경우, 메르스 확진환자와 같은 방 또는 진료, 처치, 병실에 머문 경우, 혹은 환자의 호흡기 분비물과 직접 접촉한 경우로 정의하였다 [11]. 메르스의 잠복기는 2 일에서 14 일이기 때문에 밀착접촉자의 경우 14 일 동안은 증상이 나타나는지 관찰기간을 두어야 된다. 이 기간동안 밀착접촉자로 확인된 사람은 자택, 직장, 병원 등에서 2 주간 격리조치를 받게 되었다.

2015 년 5 월 말부터 6 월 중순까지 격리조치 되고 보고된 14,999 명의 명단을 질병관리본부 역학조사과로부터 제공받았다. 이 중 조사대상지역인 서울, 경기, 충청, 강원 지역 거주자 7,313 명 중

우선순위 대상자 3,371 명에게 전화를 걸어 연구참여의사를 질문하였다. 우선순위 대상자 선정은 해당지역에 거주하는 확진환자와 확진환자의 동거인, 같은 병원환자, 간병인, 방문객으로 선정하였다. 1,694 명(50%)이 조사 참여에 동의하였고 1,679 명은 참여에 동의하지 않았다. 참여를 거부한 1,679 명 중, 65 명 (4.8%)은 욕설과 폭언을 하며 강한 조사 참여 거부 의사를 보였고, 315 명 (23.2%) 은 폭언을 하며 조사참여 거부 의사를 보였으며 568 명(41.9%)은 단순 거부 의사를 표현하였다. 409 명 (30%)은 참여가 어려웠던 사람이었으며 조사 참여 거부한 이유로는 가족의 사망, 간병, 입원 및 수술, 근무, 가족의 반대, 대상자가 아니라고 주장, 알려지길 꺼려하는 경우 등이 있었다. 314 명은 개인적인 이유로 참여하지 못했고, 8 명은 조사되지 않았다. 조사에 참여한 1,692 명 중 격리 중에 메르스 확진 받은 사람은 36 명이었고, 메르스 감염으로 최종 진단되지 않은 사람은 1,656 명이었다 (Figure 1). 본 연구는 질병관리본부 기관생명윤리위원회와 (2015-07-EXP-01-R-A) 국립암센터 의생명연구심의위원회로부터 심의를 받았다 (NCC2016-0058).

조사방법

최종 선정된 대상자는 훈련된 조사원이 웹기반 조사 질문지의 내용을 질문하고 대상자의 답변을 입력하는 방식으로 정신건강평가가 진행되었으며 조사장소는 지역 보건소에서 혈청검사와 함께 실시하였다. 조사시기는 2015 년 9 월-11 월 사이에 진행되어 메르스 격리 해제 후 4-6 개월 지난 시점에 조사가 이루어졌다.

조사내용

조사내용은 격리당시 발열, 기침, 설사 등 메르스 관련 증상의 유무, 의식주 관련된 음식과 물이 충분하였는지, 목욕할 수 있었는지, 생활필수품이 구비되었었는지 여부를 질문하였고, 전화통화, 문자나 이메일, 인터넷을 사용하였는지 여부 등을 후향적으로 조사하였다. 격리상태는 병원에서 격리된 경우, 자택이나 호텔 등에 혼자 격리, 가족과 함께 자택에 격리되었는지에 대해 후향적으로 조사하였다. 성별, 직업 등의 일반정보와 정신과적 질환의 과거력, 신체적 질환의 과거력, 메르스로 인한 의료비용지출과 재정손실 여부와 손실 상세 내역을 조사하였다. 격리시점의 상태분노감과 불안증상을 후향적으로 측정하였고, 격리해제 후 4-6 개월 시점인 조사 당시의 상태 분노감과 불안증상을 측정하였다.

조사도구

분노감

분노감 측정도구는 Spielberger 가 개발한 상태-특성 분노감 표현 척도(State-Trait Anger Expression Inventory, STAXI) 중 상태분노감 (10 문항)을 Chon 등이 한국판(STAXI-K)으로 개발한 것을 사용하였다 [12, 13]. 문항 형식은 Likert 4 점 척도로 '전혀 그렇지 않다' 1 점에서 '거의 언제나 그렇다' 4 점까지로 구성되었고, 점수가 높을수록 그 특성이 높음을 의미한다. 점수의 범위는 10 점-40 점으로 본 연구에서는 분노감의 cut-off 를 14 점으로 정의하였다. 본 연구에서 내적일치도는 (Cronbach alpha)는 0.94 였다.

불안증상

불안증상은 범불안증상장애 평가도구인 Generalized anxiety disorder 7-item scale (GAD-7)을 이용하였다 GAD-7 은 정신장애진단편람 4 판 (Diagnostic and statistical Manual of Mental disorder, 4th

edition) 에 포함된 범불안증상장애 9 개 문항과 현존하는 불안증상척도에 포함된 4 개 문항을 선정하여 총 13 개 문항 중 문항상관도가 높은 7 개 문항을 선정하여 개발된 자가보고 범불안증상장애 선별도구이다 [14]. 메르스 격리 당시와 격리해제 후 4-6 개월 시점에서 7 가지 항목을 얼마나 자주 느꼈는지를 질문하였다. 4 점 likert 척도로 전혀 방해 받지 않음 (0 점), 며칠 동안 방해받음, 절반 이상 방해 받음, 거의 매일 방해 받음 (3 점)으로 점수가 높을수록 불안증상이 높음을 의미한다. 점수의 범위는 0-21 점으로 5-9 점일 경우 경도의 불안증상, 10-14 점일 경우 중등도의 불안증상, 15 점 이상일 경우 고도의 불안증상으로 평가할 수 있고, 본 연구에서는 10 점 이상을 절단점으로 하여 중등도 이상의 불안증상 증상이 있을 경우 불안증상군으로 분석하였다. 본 연구에서 내적일치도는 (Cronbach alpha)는 0.95 였다.

자료처리 및 통계분석방법

격리자 중 메르스로 확진 받은 사람들은 메르스 확진자로 메르스 감염으로 최종 진단되지 않은 사람은 메르스 격리자로 명명하였다. 연구대상자의 일반적인 정보와 격리기간 중 격리상태와 생활 환경은 메르스 확진자와 메르스 격리자로 나누어 빈도와 백분율로 제시하였고, 두 군간의 빈도의 차이는 chi-square 분석을 하였다. 격리 당시와 격리해제 후 4-6 개월 시점에서 불안증상과 분노감은 메르스 확진자와 메르스 격리자로 나누어 빈도와 백분율을 제시하였고, 두 군간의 빈도의 차이는 chi-square 분석을 하였다. 격리해제 후 4-6 개월 시점의 불안증상 및 분노감 증상 경험의 위험요인과 보호요인은 SAS 의 proc Genmod 를 이용하여 분석하였고, 성별과 연령을 보정한 RR (95% CI)를 제시하였다. 전체 대상자 중 접촉의 강도가 높은 사람을 우선하여 조사하였기 때문에 격리자에서 불안증상, 분노감 유병률이 어느 정도 과대추정 되었는지 파악하기 위해 우선

순위 정보가 확인된 1032 명 (60%) 자료를 토대로 밀착접촉자 우선 순위에 따라 불안증상 및 분노감의 유병률 차이가 있는지 chi-square 분석을 하였다. 강한 분노감으로 인하여 연구에 미참여했을 가능성이 있어서 참여자의 불안증상, 분노감 유병률이 과소추정되었을 가능성을 알아보기 위해 미참여자 거부사유를 분석하였다. 모든 통계분석은 SAS 9.3 version (SAS inc. Cary, NC)을 이용하여 분석하였고, 양측검정 유의수준 0.05 이하일 경우 통계적으로 유의한 것으로 평가하였다.

결과

메르스 격리자의 경우, 944 명 (57%)이 여성이었고 795 명 (48%)이 무직이었다. 33 명 (2.0%)이 정신과적 질환의 과거력이 있었고 14 명 (38.9%)이 신체적 질환의 과거력이 있었다. 195 명 (11.8%)이가 메르스로 인한 의료비용지출이 있었다고 보고하였고 173 명 (10.5%)이 메르스로 인한 매출감소 등 재정손실이 있었다고 보고하였다. 감염경로로는 같은 병원환자가 33.3%로 가장 높았고, 방문객 26,7%, 병원종사자 16.1%, 환자가족 9.9% 순이었다 (Table 1).

메르스 격리자에서 8.5%가 발열, 7.0%가 기침, 4.1%가 설사를 경험한 것으로 나타나 메르스 격리자에 비해 메르스 확진자가 발열, 기침, 설사와 같은 메르스 증상 경험을 더 많이 경험하였다 (P<.001). 격리 당시 의식주 지원여부는 메르스 격리자 87.3%가 격리기간 동안 음식과 물이 충분하였다고 하였고 96.9%가 격리시간 중에 목욕할 수 있었다고 하였으며 97%가 격리 기간 동안 생활필수품이 구비되어 있었다고 보고하여 메르스 확진자에 비해 격리자에서 격리 기간 동안 의식주 지원 받은 비율이 더 높았다 (P<.001). 격리기간 중 전화통화, 문자 혹은 이메일, 인터넷 사용과 같은 사회활동에 있어서는 메르스 확진자와 메르스 격리자 간의 차이가 없었다. 격리장소는 메르스 확진자의 91.7%가 병원에, 8.3%가 가족과 함께 자택에 격리된 반면, 메르스 격리자의 68.4%

가 가족과 함께 자택에, 6.3%가 병원에 격리 되어 메르스 확진자와 격리자 간의 격리장소에 차이가 있었다 (P<.001). (Table2)

메르스 확진자에 있어서 격리시점의 불안증상 유병율은 47.2% (95%CI: 30.9-65.3%), 분노감 유병율은 52.8% (95%CI: 36.5-69.1%) 였고, 4-6 개월 시점이 불안증상 유병율은 19.4% (95%CI: 6.5-32.3%), 분노감 유병율은 30.6% (95%CI: 15.6-45.7%) 이였다. 메르스 격리자에 있어서 격리시점의 불안증상 유병율은 7.6% (95%CI: 6.3-8.9%), 분노감 유병율은 16.6% (95%CI: 14.8-18.4%) 였고, 4-6 개월 시점이 불안증상 유병율은 3.0% (95%CI: 2.2-3.9%), 분노감 유병율은 6.4% (95%CI: 5.2-7.6%) 이였다.

성별과 연령을 보정한 후 격리해제 4-6 개월 지난 시점에서 불안증상 및 분노감을 경험하는 위험요인으로는 격리 당시 메르스 증상이 있었거나 구호품 보급이 충분하지 않았거나 이메일, 문자, 인터넷 등을 사용하였거나 정신과 질환의 과거력이 있었던 경우, 재정 손실이 있었던 사람이었다 (Table 4).

우선순위 정보가 확인된 1032 명 중 0 순위는 27 명 (2.6%), 1 순위 514 명 (49.8%), 2 순위 60 명 (5.8%), 3 순위 368 명 (35.7%), 4 순위 63 명 (6.1%) 였다. 메르스 접촉자에서 노출 강도 순위에 따른 불안증상 및 분노감의 차이를 알아본 하위군 분석에서 1, 2, 3, 4 순위에 따른 분노감 경험율은 각각 18.1%, 16.7%, 15.2%, 6.3% (*P for trend* < 0.001)로 우선순위에 해당될수록 분노감 경험율이 높아지는 경향성이 있었으며 1, 2, 3, 4 순위에 따른 불안증상 경험율은 각각 31.7%, 28.3%, 22.8%, 20.6%로 (*P for trend* < 0.001)로 우선순위에 해당될수록 불안증상증상 경험율이 높아지는 경향성을 보였다 (Figure 2).

고찰

메르스의 잠복기는 평균 5 일 (2 일-14 일) 이기 때문에 메르스 바이러스에 노출된 후 짧게는 2 일, 길게는 14 일정도 지난 후 증상이 발생하므로 밀착접촉자의 경우 14 일 동안은 증상이 나타나는지 관찰기간을 두어야 한다.

메르스 전파가 확산되고 있었던 2015년 6월 10일 기준으로, 전국 17개 시도에 자택 격리자가 있었다. 대규모 감염이 발생한 병원이 있었던 지역에서는 격리자가 500명에 육박하였고, 마을 전체가 격리 대상자였던 지역도 있었다. 당시 전국 자택 격리자 수는 3000명에 달하였고, 하루 200-300명씩 자택 격리자가 늘어나는 추세를 보이고 있었다.

메르스가 빠른 속도로 한국사회에 전파되면서 격리조치를 받게 된 사람들은 치사율 20%을 넘는 감염성 질환에 대한 감염 공포와 불안감, 격리 자체에서 오는 사회적 고립감이 컸을 것이고, 다른 가족이 함께 생활하는 공간에서 격리조치가 이루어 질 경우는 가족에게 감염시킬지 모른다는 불안감 또한 높았을 것으로 여겨진다. 또한 메르스 전파를 차단하기 위하여 보건소 직원이 격리상황을 감시하고 있었기 때문에 자신의 격리 상태를 이웃들이 알게 되어 낙인이 되지 않을까에 대한 불안감도 높았을 것으로 보인다.

메르스 유행 당시 일반대중의 80.2%가 자신이 메르스에 감염될까봐 두려웠다고 보고하였고, 46%는 정서적인 고통을 겪고 있다고 보고하였다. 두려움에 대한 확률을 높이는 것으로는 대중교통 이용 우려, 외출의 지장, 국가로부터 보호받지 못한다는 인식, 통제할 수 없는 상황에 대한 무력감, 감염에 대한 두려움과 공포였다 [10].

격리 당시, 불안증상 유병율은 메르스 확진자의 경우 47.2% (95%CI: 30.9-63.5%), 메르스 격리자의 경우 7.6% (95%CI: 5.3-8.9%)였다. 격리해제 후 4-6 개월 지난 시점의 불안 증상 유병율은 메르스

확진자의 경우 19.4% (95%CI: 6.5-32.3%), 메르스 격리자에서는 3.0% (95%CI: 2.2-3.9) 였다. 불안증상의 유무는 GAD-7 으로 측정한 불안증상점수에서 중등증 이상 불안증상이 있는 경우를 불안증상이 있는 것으로 정의하였다 (GAD-7≥10). 중등증 이상의 불안증상 의미는 지난 3 개월 동안 평균 10.7-16.8 일 동안 불안증상으로 인해 일상생활에 어려움을 겪으며 불안증상으로 인해 평균 2.2-2.4 회 병의원을 방문해야 할 정도의 불안증상을 의미한다 [14]. GAD-7 절단점 10 점이상을 불안증상이 있는 것으로 평가했을 때 일반인구 집단의 유병율은 약 3.3% 내외로 평가되었는데 이는 2011 년 수행했던 정신역학실태조사에서 구조화된 면담도구인 CIDI (Composite International Diagnostic Interview)로 평가한 지역사회 불안장애 유병율 6.8%에 비해 훨씬 낮은 유병율을 보이고 있다 [15]. 이는 GAD-7 의 절단점인 10 점 이상을 불안증상이 있는 것으로 평가할 때 실제 불안 증상 유병율을 과소추정 할 가능성이 있다고 볼 수 있다.

2014 년 4 월 16 일에 있었던 세월호 사건 이후 4-6 개월이 지난 시점에 안산지역과 경기지역의 불안증상을 GAD-7 으로 측정하였을 때, 안산지역 주민의 6.4%와 경기지역 주민의 3.3%가 중등증 이상 불안증상을 경험한 것으로 나타났다 [16]. 이 결과와 비교해 볼 때, 메르스 격리자 경우 격리해제 후 4-6 개월 지난 시점에는 불안증상 정도가 정상인 수준으로 회복된 것처럼 보이지만, 1 순위에 해당되는 사람들에 있어서 격리 시점에 불안증상을 보였던 사람들 중 19%가 격리해제 후 4-6 개월 시점에도 불안증상이 지속되는 것으로 나타나 격리로 인한 불안이 사실 정상화 되었다고 보기 어렵다.

분노감 유병율은 격리시점에 16.6%, 격리해제 후 4-6 개월 시점에 6.4%로 나타났다. 분노감은 상태분노를 측정하는 것으로 이는 기질적인 분노감 성향을 가지고 있지 않은 사람이 스트레스 상황에서 경험하는 일시적인 분노감을 측정하며 스트레스 사건이 해소됨으로써 시간에 따라 달라질

수 있는 감정이다. 분노감의 유무는 상태분노 척도 STAI 총점이 14 점 이상인 경우로 정의하였는데 STAI 척도에서 14 점 이상은 중등증 이상 우울증상이 28.1%, 자살생각 33.6%, 알코올 남용 39.1%를 보이는 교정시설 수감자의 평균 분노 점수를 의미한다 [17].

메르스 격리자 중 격리 당시 분노감이 있었던 사람 중 28.1%가 격리해제 후 4-6 개월 시점에서도 분노감이 지속되었고, 노출중증도 1 순위 해당된 사람들의 경우, 50%가 격리 해제 후 4-6 개월 시점까지 분노가 지속되는 것으로 나타났다. 노출중동도 순위에 따라 분노감 유병율이 1 순위 위주로 선정한 본 조사에서는 다소 과대추정되었을 가능성이 있으나 조사에 참여하지 않은 사람 중 30%가 욕설이나 폭언 등 분노감정을 표출하며 조사 참여를 거부한 경우임을 반영하였을 때, 본 조사 결과 나타난 메르스 격리자에서 분노감은 과소추정되었을 가능성이 있다.

격리 당시 격리자에게는 대한적십자사와 지역보건소에서 4 인 가족이 1 주일간 사용가능한 쌀, 물, 라면, 생활용품 등이 공급되었다. 그러나 모든 격리자에게 메르스 격리 시점에 구호품이 제공되지 않은 경우도 있어서 일상생활에 필요한 물품을 적절한 시점에 제공받지 못했을 때 불안증상과 분노감이 컸을 것으로 보인다. 전체 메르스 격리자 중 약 50%에서 구호품을 제공 받았는데 본 연구에 참여한 조사 대상자 경우에는 약 98%가 구호품을 받은 것으로 나타났다. 이는 조사대상자 선정에 있어서 상위우선순위 대상자를 조사에 포함시킨 이유 때문으로 여겨진다.

정신과 질환의 과거력이 있었던 경우, 격리 해제후 4-6 개월 시점에 불안증상과 분노감 위험이 높았었는데 정신과적 질환의 발생이 대뇌의 신경전달물질 이상으로 발생하기 때문에 정신과적 질환의 과거력이 있는 경우 감정중추와 관련된 불안증상 및 분노감 조절이 취약했을 것으로 보인다 [18]. 이는 트라우마 상황에서 정신과 질환의 과거력이 있는 사람에 대한 특별한 조치가 필요함을 시사하고 있다.

재난 초기에 경험한 불안증상, 분노감, 공격성 등은 초기에 재난으로 인한 정신건강 관리 대응을 해 줌으로써 만성화 되는 것을 막을 수 있다 [19]. 하지만 초기에 적절한 정신건강 관리를 받지 못했을 경우 만성화되어 장기적인 회상후 스트레스 장애로 이행하게 되기 때문에 불안증상과 분노감을 파악하여 적절한 중재를 통해 단기에 증상 개선을 위한 노력을 해야 한다.

격리 기간 동안 구호품은 시의 적절하게 제공 되어야 하며, 격리 기간 동안 증상에 대한 정보 제공과 격리해제 후 증상이 있었던 사람들에 대해서 심리지원이 필요하다. 재정손실 파악 및 신속한 지원이 이루어져야 하며, 정신과적 질환의 과거력이 있는 경우는 재난으로 인한 정신과적 증상 호소 가능성이 높으므로 이에 대한 파악 및 심리지원이 필요하다. 증상이 지속되는 사람들에 대해서는 의학적 관리 프로그램이 제공될 필요가 있다. 재난으로 인한 불안증상, 분노감, 공격성 등은 재난 초기에 적절한 정신건강 관리 대응을 해 줌으로써 만성화 되는 것을 막을 수 있기 때문에 신속한 대응과 개별적인 필요를 충족시켜 줌으로써 장기적인 외상 후 스트레스 장애로 이행하게 되는 것을 막을 수 있고, 불안증상과 분노감을 파악하여 적절한 중재를 제공하는 노력을 해야 한다. 메르스 환자와의 접촉으로 인해 2 주간 격리된 사람들은 격리 당시 높은 불안증상과 분노감을 호소하였고 격리해제 후 4-6 개월 시점에도 정신건강 영향이 관찰되었다.

참고문헌

1. Chua S, Cheung V, Cheung C, McAlonan G, Wong J, Cheung E, Chan M, Wong M, Tang S, Choy K, Wong M, Chu C, Tsang K. Psychological Effects of the SARS Outbreak in Hong Kong on High-Risk Health Care Workers. Can J Psychiatry 2004;49:391–393.
2. Mak I, Chu C, Yiu M, Ho S, Chan V. Risk factors for chronic post-traumatic stress disorder (PTSD) in SARS survivors. General Hospital Psychiatry 2010;32: 590–598.
3. Acierno R, Ruggiero KJ, Galea S, Resnick H, Koenen K, Roitzsch J, de Arellano M, Boyle J, Kilpatrick D. Psychological sequelae resulting from the 2004 Florida hurricanes:implications for postdisaster intervention. American Journal of Public Health 2007;97(Suppl 1): S103–S108.
4. Mason, V., Andrews, H., & Upton, D. The psychological impact of exposure to floods. Psychology, Health & Medicine 2010;15:61-73.
5. Norris, F. H. (2005). Range, magnitude and duration of the effects of disasters on mental health: Review update 2005. Retrieved January 6, 2009.
6. Galea, S., Nandi, A., & Vlahov, D. The epidemiology of post-traumatic stress disorder after disasters. Epidemiologic Reviews 2005;27: 78-91.
7. Evans S, Giosan C, Patt I, Spielman L, Difede J. Anger and its association to distress and social/occupational functioning in symptomatic disaster relief workers responding to the September 11, 2001, World Trade Center disaster. Journal of Traumatic Stress 2006;19:147–152.

8. Jakupcak M, Conybeare D, Phelps L, Phelps L, Hunt S, Holmes HA, Felker B, Klevens M, McFall ME. Anger, hostility, and aggression among Iraq and Afghanistan war veterans reporting PTSD and subthreshold PTSD. Journal of Traumatic Stress 2007;20: 945–954.
9. 이선영. 대형재난사고 유가족의 생활경험연구. 한국사회복지교육 2011;15:109-144.
10. 이동훈, 김지윤, 강현숙 (2016). 메르스 감염에 대한 일반대중이 경험한 두려움과 정서적 디스트레스에 관한 탐색적 연구. 한국심리학회지: 일반. Vol. 6. 327-353.
11. Korea Centers for Disease Control and Prevention. [Internet]. Cheongju: Korea Centers for Disease Control and Prevention; 2015 [cited 2015 Jul 7]. Available from: http://www.mers.go.kr/mers/html/jsp/main.jsp/menu_F1/content_F2.jsp
12. Spielberger, C. D.(1983). Manual for the State-Trait Anxiety Inventory. Palo alto, CA, Consulting Psychologist Press. P.10.
13. Chon KK, Hahn DW, Lee CH, Spielberger CD. Korean adaptation of the state-trait anger expression inventory: anger and blood pressure. Korean J Health Psychol 1997;2:60-78.
14. Spitzer RL, Kroenke K, Williams JB, Löwe B. A brief measure for assessing generalized anxiety disorder: the GAD-7. Arch Intern Med 2006;166:1092-1097.
15. 정신역학실태조사, 2011, 보건복지부
16. Yang H, Cheong H, Choi B, Shin M, Yim H, et al. Community mental health status six months after the Sewol ferry disaster in Ansan, Korea. Epidemiol Health 2015; 37: e2015046.

17. Park J, Kim Y, Lee S. Mental health status of prospners in correctional institute. J Korean Neuropsychiatr Assoc 2013;52:454-462.

18. Yehuda R. Current status of cortisol findings in post-traumatic stress disorder: The role of neural networks. Psychiatr Clin North Am 2002;25:341-368.

19. Adams RE, Boscarino JA and Galea S (2006) Social and psychological resources and health outcomes after the World Trade Center disaster. Social Science & Medicine 62: 176–188.

20. Spitzer RL, Kroenke K, Williams JB, Löwe B. A brief measure for assessing generalized anxiety disorder: the GAD-7. Arch Intern Med 2006;166:1092-1097

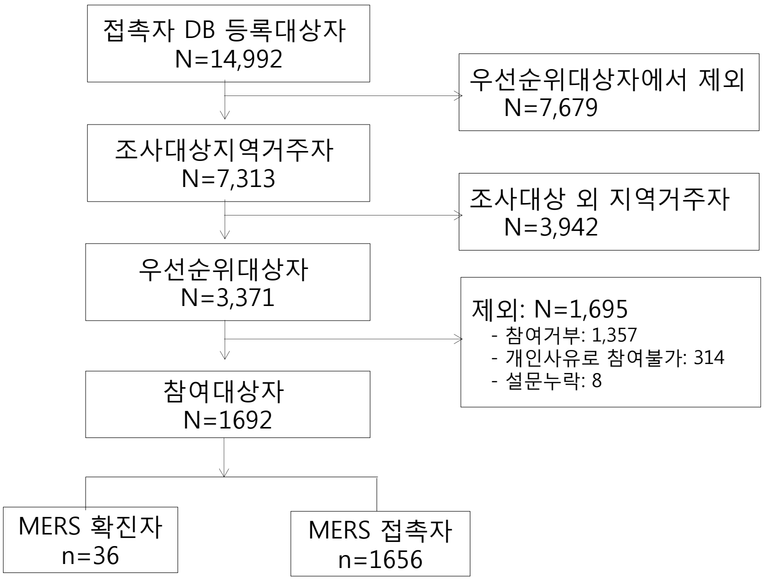

Figure 1. Flow diagram of selection of the participants

Table 1. General characteristics of 1,692 among MERS-cases and isolated people

	MERS cases	Isolated people	P-value

	(n=36)	(n=1656)	
Sex			0.401
Male	18 (50.0)	712 (43.0)	
Female	18 (50.0)	944 (57.0)	
Age	52.3 ± 15.0	43.9 ± 19.2	0.009
Job			0.471
Self-employed	6 (16.7)	174 (10.5)	
Employed	13 (36.1)	687 (41.5)	
Unemployed	17 (47.2)	795 (48.0)	
History of mental disease			<.001
Yes	8 (22.2)	33 (2.0)	
No	28 (77.8)	1623 (98.0)	
History of physical disease			0.022
Yes	14 (38.9)	375 (22.6)	
No	22 (61.1)	1281 (77.4)	
Medical cost expenditure			<.001
Yes	20 (55.6)	195 (11.8)	
No	16 (44.4)	1461 (88.2)	
Financial loss			0.230
Yes	6 (16.7)	173 (10.5)	
No	30 (83.3)	1483 (89.5)	
Infection routes			<.001
Same ward patients	20 (55.7)	551 (33.3)	
Patients' family	0 (0.0)	164 (9.9)	
Care takers	6 (16.7)	30 (1.8)	
Visitor	4 (11.1)	442 (26.7)	
Hospital workers	4 (11.1)	267 (16.1)	
Others	2 (5.7)	202 (12.2)	

Data were presented n (%) or Mean ± SD

Table 2. Living status and environmental situation during the isolation

	MERS cases	Isolated people	P-value

Variables	(n=36)	(n=1656)	
MERS related symptoms			
Fever	28 (77.8)	141 (8.5)	<.001
Cough	20 (55.6)	116 (7.0)	<.001
Diarrhea	16 (44.4)	67 (4.1)	<.001
Food, clothes, and house supplies			
Sufficient food and water	23 (63.9)	1446 (87.3)	<.001
Able to take bath	27 (75.0)	1600 (96.6)	<.001
Having self care products	29 (80.6)	1606 (97.0)	<.001
Social networking activities			
Making phone calls	31 (86.1)	1354 (81.8)	0.503
Texting or e-mailing	1 (2.8)	111 (6.7)	0.510
Using the internet	2 (5.6)	231 (13.9)	0.218
Isolation environment			<.001
Hospital	33 (91.7)	104 (6.3)	
Alone (in home or hotel)	0 (0)	419 (25.3)	
With family	3 (8.3)	1133 (68.4)	

Table 3. Prevalence of anxiety symptom and anger during isolation and after 4-6 months from release

Variables	MERS cases (n=36)	Isolated people (n=1656)
In isolation		
Anxiety	47.2 (30.9-63.5)	7.6 (6.3-8.9)
Anger	52.8 (36.5-69.1)	16.6 (14.8-18.4)
After 4-6 months from release		
Anxiety	19.4 (6.5-32.3)	3.0 (2.2-3.9)
Anger	30.6 (15.6-45.7)	6.4 (5.2-7.6)

Table 4. Relative risk of anxiety and anger during the period between 4-6 months after release from the isolation

	MERS cases (n=36)		Isolated people (n=1656)	
Variables	Anxiety (n=7)	Anger (n=11)	Anxiety (n=49)	Anger (n=106)
MERS related symptoms				
Fever (Ref=no)	1.7(0.3-9.1)	1.5 (0.3-9.2)	1.8 (1.1-3.0)	2.4 (1.4-4.1)
Cough (Ref=no)	1.8 (0.4-7.7)	1.6 (0.4-6.9)	3.1 (2.0-5.0)	3.8 (2.3-6.3)
Diarrhea (Ref=no)	3.7 (0.8-18.0)	4.1 (0.8-20.6)	5.3 (3.1-9.0)	6.7 (3.8-11.8)
Food, clothes, and house supplies				
Sufficient food and water (Ref=yes)	5.9 (0.9-34.5)	3.8 (0.8-18.6)	3.2 (2.2-4.7)	3.2 (2.0-5.0)
Able to take bath (Ref=yes)	3.3 (0.6-17.8)	1.1 (0.2-5.7)	2.1 (1.1-4.2)	2.9 (1.3-6.4)
Having self care products (Ref=yes)	1.0 (0.2-5.9)	2.1 (0.4-12.4)	2.7 (1.3-5.3)	3.1 (1.4-6.7)
Social networking activities				
Making phone calls (Ref=no)	1.3 (0.1-13.6)	1.7 (0.1-21.4)	1.1 (0.7-1.7)	1.1 (0.7-1.8)
Texting or e-mailing (Ref=no)	NA		2.2 (1.3-3.7)	2.2 (1.2-4.0)
Using internet (Ref=no)	NA		1.8 (1.1-2.7)	1.9 (1.1-3.1)
History of mental disease (Ref=no)	10.7 (1.1-109.6)	2.9 (0.5-17.3)	5.3 (2.5-11.0)	4.0 (1.7-9.5)
History of physical disease (Ref=no)	2.4 (0.6-10.2)	3.4 (0.7-15.6)	1.8 (1.3-2.6)	1.5 (0.9-2.4)
Medial cost expenditure (Ref=no)	5.5 (1.0-30.7)	1.6 (0.3-7.7)	3.7 (2.5-5.5)	5.5 (3.5-8.5)
Financial loss (Ref=no)	3.3 (0.7-15.7)	1.2 (0.3-5.6)	1.9 (1.4-2.6)	1.6 (1.1-2.3)

All RRs (95%CIs) were presented after adjusting for age and sex

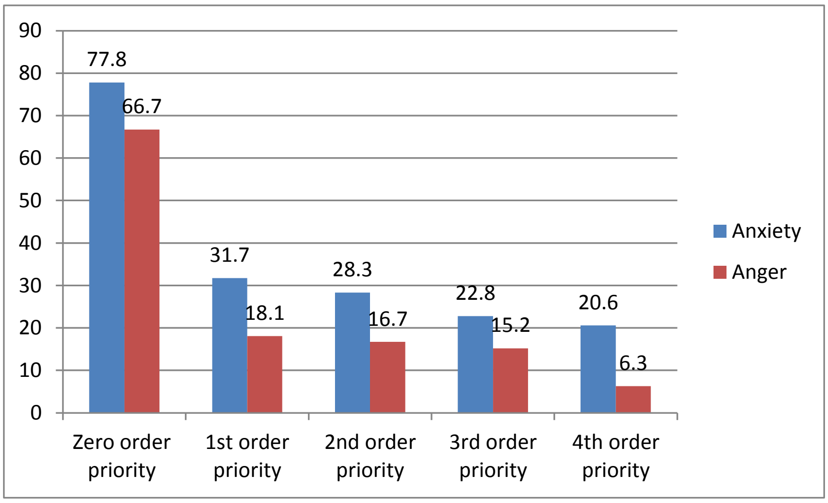

Figure 2. Prevalence of anxiety and anger according to the exposure intensity in MERS infection
